# Supplementary material for: Ethnoveterinary Practices and Ethnobotanical Knowledge on Plants Used against Cattle Diseases among Two Communities in South Africa
Source: Plants (Basel). 2022 Jul 5;11(13):1784. doi: 10.3390/plants11131784 (PMC9268905; doi:10.3390/plants11131784)
Supplement: Supplementary file 1 [file plants-11-01784-s001.zip › plants-1778070 supplementary.pdf]

Supplementary Table S1. Demographic information of the study areas.

Population characteristics of the study areas	Category	Dinokana	Gopane
1. Population		26409	10035
2. Gender	• Male	46.8	46.7
	• Female	53.2	53.3
3. Number of households		6543	2691
4. Language	• Setswana	92.4%	87.9%
	• English	2.6%	5.7%
	• IsiNdebele	1.2%	1.5%
	• isiZulu	1.3%	1.1%
	• IsiXhosa	0.3%	1.6%
	• Other	2.2%	2.2%
5. Ethnicity	• Black African	99.4%	99.6%
	• Coloured	0.2%	0.0%
	• Indian/Asian	0.2%	0.2%
	• White	0.1%	0.0%
	• Other	0.1 %	0.1 %
